# Elementary school compliance with a state recess minimum requirement by racial and geographic factors: a cross-sectional study

**DOI:** 10.1186/s12966-025-01730-x

**Published:** 2025-03-28

**Authors:** Erin K. Howie, Samantha M. Harden, Daheia J. Barr-Anderson, Christopher R. Long

**Affiliations:** 1 Department of Health, Human Performance and Recreation, University of Arkansas, Fayetteville, AR USA; 2 Department of Human Nutrition, Foods, and Exercise, Virginia Tech, Blacksburg, VA USA; 3 Department of Obstetrics/Gynecology, Department of Family and Community Medicine, Virginia Tech Carilion School of Medicine, Roanoke, VA USA; 4 Department of Applied Physiology, Health, and Clinical Sciences, University of North Carolina at Charlotte, Charlotte, NC USA; 5 Center for Nutrition & Health Impact, Omaha, NE USA

**Keywords:** Physical activity, Policy, Implementation, Health disparities

## Abstract

**Background:**

Recess is a part of school-based physical activity promotion offered worldwide with equitable recess access a social justice issue. From a policy perspective, in the U.S. few states currently require elementary school recess and little is known about its implementation. The purpose of this study was to determine the current implementation of one state system as a case study to investigate minimum recess requirement and to compare the implementation between school geographic and racial factors.

**Methods:**

A cross-sectional, observational study of the implementation of one state’s minimum daily recess requirement of 40-minutes recess was conducted during the 2023–2024 academic year. A school audit of provided recess time was conducted of all public elementary schools in Arkansas through an online search of bell schedules, a survey sent to principals and physical education teachers, and phone call surveys to school offices. Key demographic and geographic features of the schools included enrollment data (e.g., race, grade, and % Free-and-Reduced Lunch composition), rurality, and region.

**Results:**

Recess information was obtained from 384 (73%) of 526 eligible schools with an average student enrollment of 398 students (SD 154), 19.8% (SD 27.9) Black student enrollment, and 63.8% (SD 20.0%) students receiving free-and-reduced lunch. 306 (85.5%) schools met recess requirements. There were no differences in meeting recess requirements by rurality. Of schools with higher Black student enrollment (≥ 25% Black enrollment), 75.3% met recess requirements compared to 89.5% in schools with lower Black enrollment (< 25% Black enrollment, OR 0.36, 95%CI: 0.16, 0.78, *p* =.010). There were differences in survey-reported available playground spaces and equipment between by meeting recess requirements and Black student enrollment (*p* <.05).

**Conclusions:**

Schools in a state with a 40-minute daily recess requirement reported high compliance with the state policy. However, students in schools with higher Black student enrollment were less likely to meet the 40-minute recess requirement, and thus strategies are needed to ensure all students have access to recess opportunities. Ensuring equal access to recess through wide-reaching place-based and policy-based strategies may be a step in reducing health and education disparities, especially among populations where disparities are greatest.

**Supplementary Information:**

The online version contains supplementary material available at 10.1186/s12966-025-01730-x.

## Background

Recess, a regularly scheduled period in the school day for physical activity and play [1], has been associated with many positive benefits including physical, mental, socioemotional and educational outcomes [2] (e.g., behavior and academic achievement [3]). One potential explanation for the positive effects of recess on student outcomes is the participation in physical activity during recess [2], as physical activity has been associated with these numerous outcomes [4]. Studies using nationally representative data have found that students offered at least 30 min of recess have higher levels of physical activity reported by parents [5, 6]. However, when examining device-measured physical activity, there were no differences in physical activity or fitness between those with high recess provision of at least 30 min daily compared to low or no recess provision [6]. These inconsistencies may be explained by gaps in recess implementation and differences in recess quality.

Because of these benefits of recess for student developmental outcomes, recess is a practice implemented worldwide [7], and included as a key part of physical activity promotion in schools globally [8]. Cross-cultural comparisons have highlighted similarities and differences in recess practices internationally including the duration and frequency of scheduled recess periods [9, 10]. In the U.S., the Centers for Disease Control and Prevention (CDC) recommends a minimum of 20 min of recess daily for elementary students [1]. There is currently no surveillance system to regularly determine the amount of recess provided in the United States and estimates range widely. A study combining past national surveillance systems from between 2012 and 2016 estimated that 65 to 90% of students receive at least 20 min of recess [11]. However, studies report recess amounts inconsistently and many of the studies used guardian-reported recess provision, which may not accurately represent recess provision at the school-level making studies difficult to compare. For example, the 2012 National Youth Fitness Study, which uses guardian-reported recess time, found 35% of participants to have no to low recess provision of less than 15 min per day [6]. In contrast one more recent national survey of 559 schools during the 2019–2020 school year found that 60% of schools reported providing daily recess of 30 min or more [12]. A study of recess implementation among low-income schools in California found 56% of schools reported at least 20 min of daily recess [13]. A better understanding of the implementation of recess policy will help to build implementation science and evidence-based policies more broadly [14], specifically how educational policies that may influences physical activity are translated into practice [15].

Specifically, most of these national studies do not examine recess provision by racial and geographic factors. There is evidence that non-white children and children in certain geographic locations receive less recess. However, findings are mixed with the Early Childhood Longitudinal Survey Kindergarten Class of 1998–1999 reporting schools with lower socioeconomic students and larger percentage of minority populations had less recess time [16] and a more recent national survey finding no differences by race or socioeconomic status [12]. The survey of recess practices in California found that schools with higher school poverty were less likely to implement at least 20 min or more of recess, but no differences by school racial demographics [13]. Evidence suggests that rural and Black children are at increased risk for obesity and heart disease [17, 18] and thus may benefit most from increased opportunities for physical activity, such as recess. The existence of place- and race-based disparities in education and health suggests that it is worthwhile to investigate place- and race-based disparities in recess policy. Equitable access to quality recess has been advocated as a social justice issue [19] with the right to play recognized internationally by the United Nations’ Convention on the Rights of the Child [20]. More evaluation of recess implementation at the school level, by geographic and demographic factors, is needed to better understand the equitable provision of recess.

One way to potentially increase and regulate the amount of recess all students receive is through state policies requiring a minimum duration of recess. Recess policy requirements have been associated with higher levels of physical activity and better socioemotional outcomes for students [21], but the relationship between state policies and school-level implementation has been less clear. Research has found that school districts are more likely to require recess in states with legislation requiring recess, but the same study also found that schools provided more recess in states recommending and not requiring recess [22]. However, this previous study was unable to examine the effects of specific recess requirements, and did not examine recess provision by racial, socioeconomic or geographic factors. One reason for the complicated relationship between policies and student outcomes may be the policy-practice gap, as not all policies result in the intended changes to practice. In this case, not all schools may comply with recess policies, and schools out of compliance may have different student outcomes. A study of four schools in Arizona, where state policy requires two daily recess periods without specifying minimum durations, found that students in complying schools had higher physical activity than those in non-complying schools [23]. Previous research on physical activity policies suggests that policy changes do not always lead to changes in practice due to variations in implementation [15]; thus, research is needed to examine the compliance with state recess laws.

Despite potential benefits of state recess policies on student outcomes, only 10 states required a minimum amount of recess for elementary schools in 2024–2025 school year. This includes an additional three states passing recess laws in the past two years showing increased interest and action around recess legislation and a need for a stronger evidence base to support new and existing policies. Additionally, there is a wide range in the requirements of these policies [24]. For example, Arkansas requires the most amount of recess at 40-minutes daily, compared to Louisiana requiring 15 min daily. Importantly, the implementation of these policies has not been evaluated. State recess laws have the potential to increase equitable access to quality physical activity opportunities. However, policies that are not equally implemented could exacerbate health disparities. As several states continue to consider passing recess legislation, it is important to identify the potential impact of state recess legislation on recess provision for students from diverse racial and geographic backgrounds. Implementation evaluations of physical activity policies can contribute critical information on the policy to practice gap, yet no evaluation of the implementation of a state policy considering racial and geographic factors has been conducted.

Thus, the purpose of this study was to determine the current implementation of one state system’s (Arkansas) minimum recess requirement and to compare the implementation between geographically and racially diverse schools as an example of examining policy implementation among health disparate populations.

## Methods

### Study design

A cross-sectional, observational study of the implementation of Arkansas’s recess requirement (Arkansas Code Title 6. Education § 6-16-102) was conducted in March to July 2024 of the 2023–2024 academic school year. This recess requirement was implemented in the 2019–2020 school year and requires: “At least forty (40) minutes of instructional time per school day shall be used for recess during the school day for students attending public elementary schools” and that “recess shall: Consist of supervised, unstructured social time during which public school students may communicate with each other, occur outdoors when weather and other relevant conditions permit; and include without limitation opportunities for free play and vigorous physical activity, regardless of whether recess occurs indoors or outdoors.”

Biases often exist in those who respond to surveys which may hamper our understanding of health disparities [25]; thus, research staff conducted a multipronged audit of all elementary schools in the Spring (March to June) of 2024. This study utilized three methods to collect recess implementation data to ensure completeness of data through an electronic and phone school audit [26]. First, an online search was completed by research assistants to collate publicly available recess bell schedules. Second, an online survey was sent to school principals and physical education teachers when contact information was available. The survey collected information on recess provision and stakeholder perceptions. Finally, for schools without survey responses, research assistants called school offices to collect available information on recess provision.

Schools were eligible if they were public, including public charter schools, and classified as elementary including any grades kindergarten through 5th grade. Schools were excluded if they were virtual or hybrid schools, preschools, or only included middle and high school grades (6th grade and above). From the 1,198 schools obtained from the Arkansas Department of Education Data Center [27] (*n* = 1,058 public schools, *n* = 140 private), there were 526 eligible public and charter elementary schools in Arkansas during the 2023–2024 school year. The study was determined as exempt by the University of Arkansas Institutional Review Board.

### Measures

Implementation was defined as the total daily amount of recess in minutes with additional variables for the duration and frequency of recess periods.

#### Online search

Research assistants conducted a systematic search of school websites and Google to search for available recess schedules. Assistants used keywords such as “bell schedule” and searched wellness policies and parent handbooks for information. Copies of schedules were saved electronically and the duration and frequency of daily recess periods were extracted and used to calculate the total daily minutes of recess.

#### School survey

School principals and physical education teachers were asked to report the total recess duration of each grade, the number of recess sessions, and the average length of each recess period via an online survey administered through Qualtrics (Provo, UT). Principals were selected as the primary contact at the school typically responsible for setting school schedules. Physical education teachers were also included as a key physical activity and health contact. The primary recess provision question was “On average how many total minutes of recess do students at your school receive each day?” with response options of “Less than 20 minutes”, “20–29 minutes”, “30–39 minutes”, “40–49 minutes”, “50–59 minutes”, “1 hour or more per day”, or “I don’t know”. Participants were given the option to report separate amounts per grade if all students did not receive the same amount of recess. Participants were also able to provide a copy of their recess/bell schedule as an additional file. Additional closed-response questions included the provision of physical education, and the quality of recess such as supervision, indoor recess policies, playground space and equipment available, and recess withholding policies. The survey was piloted through SHAPE Arkansas, the professional organization for physical education teachers. The survey was initially emailed in late March/early April, a second email follow-up email was sent in mid-April, and a final email was sent after the end of school in late June 2024.

When schools had multiple responses (*n* = 11), the survey with complete responses was used (*n* = 1. If both surveys were complete, the principal was used as the primary respondent (*n* = 6). If completed twice by the same role, the second submission with the later date was used (*n* = 4).

#### School phone calls

Research assistants obtained primary school office contact numbers available online to contact schools without online information or complete survey responses. During school business hours, they called school offices and read a prepared script. If the staff was able and willing to participate, they were asked, “On average, how many total minutes of recess do students at your school receive each day?” They were able to answer separately for different grades. They were also asked, “How many recess periods do students receive per day”, “How long is each recess period?” and asked if they could provide a copy of their school’s recess schedule via email. If the staff who answered the phone could not provide the answers, they either forwarded on the call, provided additional contact information, or were emailed the link to the online survey. Schools were contacted up to two times. Initial phone calls were made in late April/early May, with follow-up calls made in late May 2024. Total daily minutes of recess was calculated by combining the duration of each recess period by the frequency of recess periods.

Demographic data, student enrollment and enrollment by race, for the 2023–2024 school year was obtained from the Arkansas Department of Education Data Center [28]. To examine school racial differences in recess provision, the current study focused on Black student enrollment due the large health and education disparities experienced in this population [17, 18], and it is the largest minority group in Arkansas The mean percentage of Black students among eligible schools in Arkansas is 20.3% with a median of 4.5%. To capture schools with a high percentage of black students, a 25% cutoff was used to dichotomize schools due to perceived cultural norms and perceptions with substantial white or black enrollments [29]. This cutoff has been previously used to describe schools with higher Black enrollment [30, 31], and represents a natural categorization with 373 schools (71% of eligible schools) having less than 25% of Black student enrollment, 153 (29%) schools with higher than 25% Black student enrollment, and 38 schools having 0% enrollment. The mean Black student enrollment in higher Black enrollment schools was 58.2% (SD 22.4) and 4.8% (SD 6.3) in lower Black enrollment schools. Urban/rural status was determined by county categorization from the Office of Management and Budget and the Federal Office of Rural Health Policy [32]. Regions were classified into Northwest, Northeast, Central, Southwest, and Southeast according to Arkansas Department of Health regions.

Factors that may indicate overall school quality and academic environment were included as potential confounders of recess provision. School economic disadvantage and standardized test performance were obtained from the most recent Arkansas Office for Education Policy report (2022–2023 school year) [33]. Schools were categorized by grades included. Lower elementary schools were considered as those including K-2nd grade only, upper elementary included 3rd grade and higher, and mixed included both lower and upper elementary grades. School academic achievement was also included as both growth and weighted academic achievement. Weighted achievement represents how well students performed on state annual assessments and is highly negatively correlated with school economic disadvantage. Value-added growth represents how much a student improved their score on state assessments compared to other students across the state who had similar prior test scores, with low correlation with school poverty status. Higher scores on both measures represent positive growth and higher academic achievement. School economic disadvantage was reported as the percent of students eligible for free-or-reduced lunch (%FRL).

### Statistical analysis

Schools were determined to meet recess requirements if they reported providing 40 or more minutes of recess daily. When schools reported different times for different grades (*n* = 13 on survey, *n* = 10 on phone), either the most common duration or 3rd grade if there was no majority was used to determine compliance. If the recess schedule included a time for combined lunch/recess without separating recess time, a 30-minute lunch based on average reported lunch times including transition was used with the remaining time allocated as recess. Recess implementation reported from multiple sources was compared to aid validity. When an online or submitted recess schedule conflicted with a phone or survey response, the survey or phone response was used due to potential for incomplete recess schedules which may not include all recesses or be interpreted by researchers correctly. If a conflict in meeting requirements remained, we defaulted to the school meeting requirements.

Schools with missing data either from the school audit or the school survey were compared to schools with provided information on school characteristics using t-tests or chi-squared tests. Daily recess time was summarized with comparisons made between those meeting and not meeting recess requirements on school demographics, percentage of students eligible for FRL, rural/urban location, geographic location, and school achievement metrics using t-test or chi-squared tests. Multilevel logistic regressions, accounting for clustering of schools by school district, were used to determine the odds of meeting recess requirements by rurality, region and Black student enrollment with an additional model adjusting for school total enrollment and school economic disadvantage (%FRL). Models 1 were examined separately for each independent variable, Model 2 combined the three independent variables in a single model to examine independent effects, and Model 3 adjusting for school total enrollment and school economic disadvantage (%FRL). Comparisons of survey responses were made between those meeting recess requirement and not meeting recess requirements, rural/urban status, and Black student enrollment using chi-squared tests. Analysis was conducted using Stata/IC 14.2 (College Station, TX) with statistical significance set at *p* <.05.

## Results

Recess information was obtained from 384 out of 526 eligible schools for a 73% response rate. All schools without survey or online information were contacted by phone. A total of 70 schools provided copies of their recess schedule either through the survey (*n* = 48) or direct email (*n* = 22). Schools were similar between those providing information by phone or survey as seen in Table 1, however the schools with only online recess schedules were less rural and had higher academic achievement growth. Of the schools with only online information (*n* = 26), 10 (38.5%) met or exceeded recess requirements compared to 86% of schools with survey or phone information. Thus, schools with online-only recess information were removed from the analysis, and sensitivity analyses were conducted excluding the schools with online-only information and full analyses are presented in Supplemental Content 1.

**Table 1:** Description of schools by sources of information

	Survey information (*n* = 178)	Phone Information (*n* = 180)	Online recess info only (*n* = 26)	*p*-value for chi-squared or ANOVA
School Enrollment, M students (SD)	392.2 (154.5)	403.6 (153.5)	451.4 (192.0)	0.193
Grades in School, N (%)				0.725
*Lower (K-2nd)*	12 (6.7%)	15 (8.3%)	1 (3.9%)	
*Upper (3rd grade and above)*	15 (8.4%)	16 (8.9%)	4 (15.4%)	
*Mixed (Lower and Upper grades)*	151 (84.8%)	149 (82.8%)	21 (80.8%)	
Enrollment by Race, M % (SD)				
*% Black*	19.7 (28.0)	19.8 (27.3)	14.4 (13.1)	0.616
*% Hispanic*	12.0 (13.7)	12.6 (14.6)	19.4 (21.2)	0.057
*% White*	59.5 (29.7)	59.2 (28.8)	57.1 (24.6)	0.925
*% Minority*	39.4 (29.5)	39.8 (28.5)	41.2 (24.7)	0.958
Schools with high Black enrollment, N (%)	50 (28.1%)	51 (28.3%)	4 (15.4%)	0.366
% Students receiving FRL, M (SD) ^a^	64.2 (20.6)	63.4 (19.4)	63.1 (21.4)	0.917
Schools in Rural Counties, N (%)	74 (41.6%)	70 (38.9%)	3 (11.5%)	**0.013**
Region, N (%)				0.182
*Northwest*	65 (36.5%)	68 (37.8%)	8 (30.8%)	
*Northeast*	38 (21.4%)	32 (17.8%)	3 (11.5%)	
*Central*	45 (25.3%)	55 (30.6%)	14 (53.9%)	
*Southwest*	20 (11.2%)	16 (8.9%)	0 (0%)	
*Southeast*	10 (5.6%)	9 (5.0%)	1 (3.9%)	
Charter School, N(%)	7 (4.0%)	7 (3.9%)	0 (0%)	0.586
Weighted Achievement ^a^, M (SD)	56.1 (15.0)	57.8 (15.0	60.9 (14.6)	0.247
Value-added Growth Score ^b^, M (SD)	80.4 (3.3)	80.9 (3.1)	82.1 (3.4)	**0.030**
Meeting recess requirements	153 (86.0%)	153 (85.0%)	10 (38.5%)	**< 0.001**

Note: 6 schools had information from both phone and survey and are included in the survey column, 3 schools in the survey group did not have school achievement %FRL, %minority, or academic achievement data from 2022 to 2023 OEP ^a^ performance on state standardized tests with higher scores indicating higher performance, ^b^ within student change in standardized test performance with higher scores indicating greater growth

A description of the schools, comparing those with and without recess information can be found in Table 2. A total of 178 schools had completed survey responses, 185 provided information via phone interviews, and 38 recess schedules were obtained publicly online. All schools without survey or online information were contacted by phone. Reasons for failure to obtained recess information included messages left with office or transfers to voicemails (*n* = 65), requests for an email survey (*n* = 19), inaccurate phone numbers or no answers (*n* = 14), could not give information (*n* = 13), hang-ups (*n* = 8), responded too busy to answer (*n* = 6), 15 had insufficiently detailed notes from research assistants, and 2 had online schedules publicly available but were not complete and phone information was unavailable. Recess information was obtained from schools in 71 out of 75 counties. The four counties not represented were from the Northwest Region (4 eligible schools), Northeast Region (3 eligible schools), Southwest Region (1 eligible school) and Southeast Region (1 eligible school). Of those with recess information, 38.3% were in rural counties and the average Black student enrollment was 19.4%. Schools with recess information had a lower percentage of students receiving free-or-reduced lunch (63.8% vs. 67.8%, *p* =.043). There were no statistically significant differences in total student enrollment, rural or regional locations, or school achievement measures between those with and without recess information.

**Table 2:** Comparison of those with and without recess information, mean (SD), n(%)

	Recess Info Obtained (*n* = 358)	No Recess Info (*n* = 142)	*p*-value
School Enrollment, M(SD)	397.9 (153.9)	389.1 (164.1)	0.574
Grades in School, N(%)			0.393
*Lower*	28 (7.3%)	15 (10.6%)	
*Upper*	35 (9.1%)	15 (10.6%)	
*Mixed*	321 (83.6%)	112 (78.9%)	
Enrollment by Race, M(SD)			
*% Black*	19.8 (27.9)	22.9 (29.5)	0.270
*% Hispanic*	12.3 (14.2)	13.5 (15.2)	0.420
*% White*	59.3 (29.2)	56.3 (31.6)	0.311
% *Minority*^a^	39.6 (28.9)	43.6 (31.7)	0.177
Schools with high Black enrollment, N(%)	105 (27.3%)	48 (33.8%)	0.148
% Students receiving FRL, M(SD) ^a^	63.8 (20.0)	67.8 (19.7)	**0.047**
Schools in Rural Counties, N(%)	147 (38.3%)	58 (40.9%)	0.592
Region, N(%)			0.100
*Northwest*	141 (36.7%)	47 (33.1%)	
*Northeast*	73 (19.0%)	37 (26.1%)	
*Central*	114 (26.7%)	30 (21.1%)	
*Southwest*	36 (9.4%)	16 (113%)	
*Southeast*	20 (5.2%)	12 (8.5%)	
Charter School ^a^, N(%)	14 (3.7%)	4 (2.9%)	0.679
Weighted Achievement ^a, b^, M(SD)	57.0 (15.0)	54.8 (16.1)	0.151
Value-added Growth Score ^a, c^, M(SD)	80.7 (3.2)	80.2 (3.2)	0.117

^a^ 8 schools did not have school achievement %FRL, or %minority from 2022–2023 OEP (*n* = 3 with recess info, 5 without recess info), ^b^ performance on state standardized tests with higher scores indicating higher performance, ^c^ within student change in standardized test performance with higher scores indicating greater growth

### Schools meeting recess requirements

Of the 358 schools included with complete recess information, 306 (85.5%) reported meeting or exceeding 40-minutes of recess per day. The majority reported 40-minutes of recess, with 267 (74.6%) reporting 40–49 min. Of those with less than the requirement, 40 (11.2%) reported 30–39 min, 11 (3.1%) reported 20–29 min and one (0.3%) reported less than 20 min. Of those exceeding the requirement, 17 (4.8%) reported 50–59 min and 22 (6.2%) reported 60 min or more. The majority (*n* = 225, 62.9%) reported two recess periods per day, 63 (17.6%) reported one recess period per day, 13 (3.6%) reported three or more recess periods per day, and 57 (15.9%) reported a different number of recess periods for different grade levels.

**Fig. 1:**
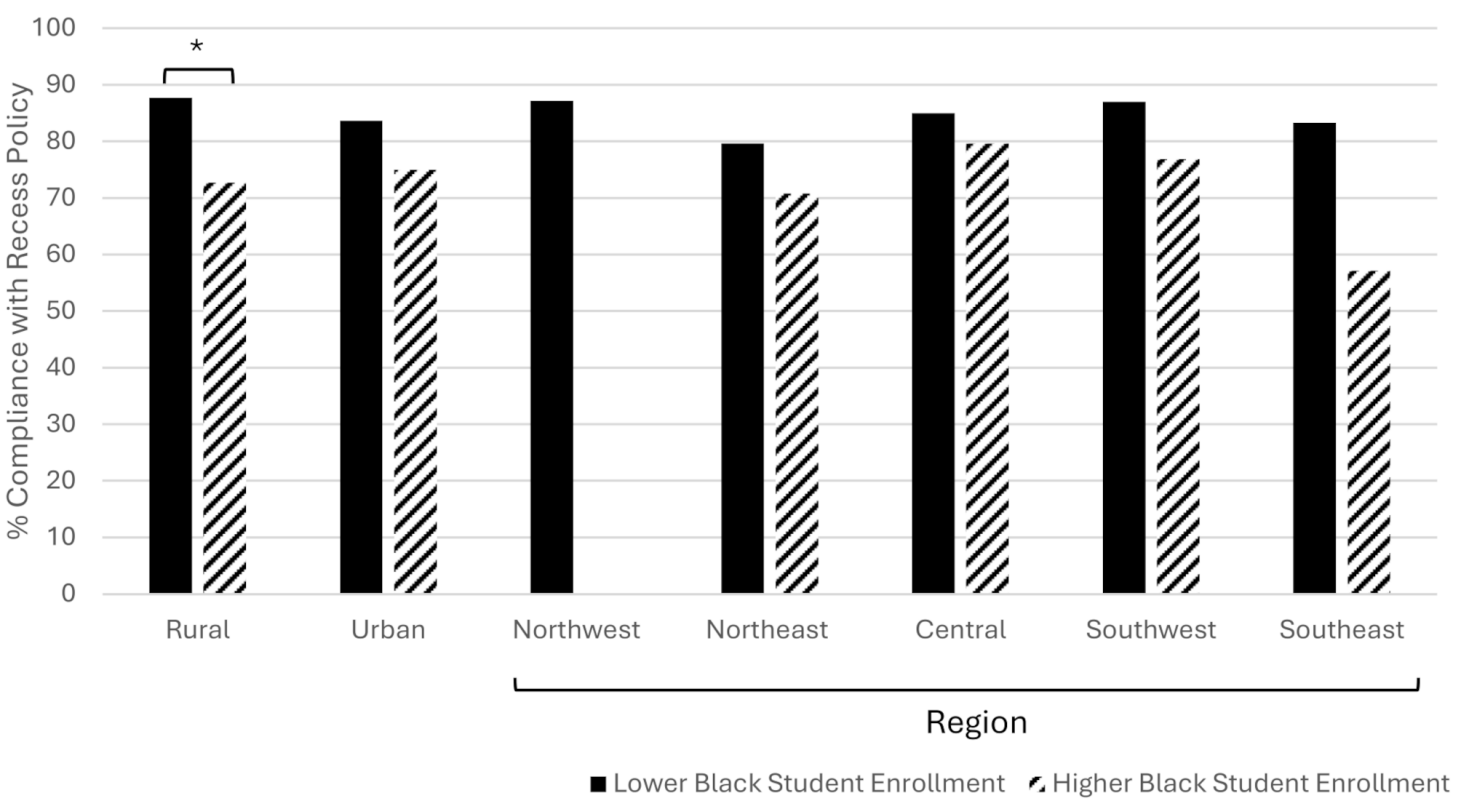
Compliance with recess policies across geographies by higher and lower Black student enrollment Note: Higher Black Enrollment (>= 25% Black student enrollment), Lower Black enrollment (<25% Black student enrollment). There were no Higher Black Enrollment schools in the Northwest Region

A comparison of school characteristics between those meeting recess requirements and those not meeting requirements can be seen in Table 3. Of schools in rural counties, 85.4% met requirements similar to 85.5% in urban counties (*p* =.980). Of schools with higher Black student enrollment, 75.3% met recess requirements compared to 89.5% in schools with lower Black student enrollment (*p* =.001). The highest percentages of schools meeting requirements were in the Northwest Region (89.5%), Central (89.0%), and Southwest (83.3%) with the Northeast (78.6%) and Southeast (68.4%) regions having the lowest percentages of schools meeting requirements. Schools meeting recess requirements had higher weighted achievement (57.8 vs. 52.1, *p* =.011 but no differences in value-added growth scores compared to schools not meeting requirements as shown in Table 3. A comparison of compliance with recess policies by geographic and demographic factors is shown in Fig. 1.

In logistic regression as shown in Table 4, schools with higher Black student enrollment were less likely to meet the recess requirements compared to those in lower Black enrollment schools (OR 0.36, 95%CI: 0.16, 0.78, *p* =.010) and the results remained when adjusting for school size, %FRL, region and rurality.

**Table 3:** School characteristics compared by meeting recess requirements

	Meeting recess requirements (*n* = 306)	Not meeting recess requirements (*n* = 52)	*p*-value
School Enrollment, M (SD)	396.4 (154.3)	406.8 (152.6)	0.650
Grades in School, N (%)			0.430
*Lower*	25 (8.2%)	2 (3.9%)	
*Upper*	25 (8.2%)	6 (11.5%)	
*Mixed*	256 (83.7%)	44 (84.6%)	
Enrollment by Race, M (SD)			
*% Black*	17.2 (25.1)	34.7 (35.8)	**< 0.001**
*% Minority*^a^	38.0 (27.5)	48.9 (35.1)	**0.013**
*% White*	60.9 (28.0)	50.2 (34.8)	**0.014**
*% Hispanic*	13.0 (14.4)	8.2 (11.6)	**0.021**
Schools with high Black enrollment, N (%)	76 (24.8%)	25 (48.1%)	**0.001**
% Students receiving FRL, M (SD) ^a^	63.0 (20.0)	68.6 (19.3)	0.061
Schools in Rural Counties, N (%)	123 (40.2%)	21 (40.4%)	0.980
Region, N (%)			**0.041**
*Northwest*	119 (38.9%)	14 (26.9%)	
*Northeast*	55 (18.0%)	15 (28.9%)	
*Central*	89 (29.1%)	11 (21.2%)	
*Southwest*	30 (9.8%)	6 (11.5%)	
*Southeast*	13 (4.3%)	6 (11.5%)	
Charter School ^a^, N (%)	10 (3.3%)	4 (7.7%)	0.133
Weighted Achievement ^a, b^, M (SD)	57.8 (14.8)	52.1 (15.5)	**0.011**
Value-added Growth Score ^a, c^, M (SD)	80.8 (3.2)	80.4 (2.9)	0.407

^a^ 8 schools did not have school achievement, %FRL, or %minority from 2022–2023 OEP (*n* = 3 with recess info, 5 without recess info), ^b^ performance on state standardized tests with higher scores indicating higher performance, ^c^ within student change in standardized test performance with higher scores indicating greater growth

**Table 4:** Multilevel logistic regression results of odds of meeting recess requirements by geographic and Racial school characteristics, *n* = 358

	OR	95%CI	*p*
Models 1: separate models for each dependent variable			
Higher Black enrollment	**0.27**	**0.11**,** 0.65**	**0.004**
Rural	1.02	0.45, 2.29	0.971
Region (reference is Northwest)			0.104
*Northeast*	0.36	0.12, 1.03	0.058
*Central*	0.97	0.31, 2.98	0.954
*Southwest*	0.57	0.15, 2.11	0.401
*Southeast*	**0.19**	**0.04**,** 0.81**	**0.025**
Model 2: combined model			
Higher Black enrollment	**0.29**	**0.10**,** 0.81**	**0.018**
Rural	2.08	0.74, 5.89	0.166
Region (reference is Northwest)			0.150
*Northeast*	0.43	0.13, 1.45	0.172
*Central*	2.52	0.65, 9.73	0.179
*Southwest*	0.72	0.16, 3.16	0.660
*Southeast*	0.32	0.05, 1.96	0.218
Model 3: combined model adjusted for school enrollment and %FRL			
School enrollment	1.00	0.997, 1.002	0.578
%FRL	1.00	0.977, 1.03	0.945
Higher Black enrollment	0.27	0.08, 0.87	0.029
Rural	2.20	0.73, 6.63	0.160
Region (reference is Northwest)			0.152
*Northeast*	0.40	0.11, 1.44	0.159
*Central*	2.56	0.64, 10.24	0.186
*Southwest*	0.68	0.15, 3.16	0.626
*Southeast*	0.29	0.04, 1.89	0.194

### Characteristics of recess

Recess characteristics reported from the survey are reported in Table 5. The most common adult supervisors were classroom teachers (95.6%) followed by para-professionals (62.4%). There were no differences between urban and rural or lower Black and higher Black enrollment schools for supervision. The most commonly reported reasons for withholding recess were student sickness (62.4%), make-up classwork (26.5%), and punishment (25.6%). The “Other” category open-ended responses included only with a note from a doctor, parent request, injury, or additional clarification that withholding is not allowed. Schools meeting recess requirements were more likely to report withholding recess for sickness (*p* =.021) compared to those not meeting recess requirements. Schools with high Black enrollment reported lower withholding for academic reasons of making up classwork (*p* =.034), homework (*p* =.044), or taking a test/quiz (*p* =.027).

All schools reported having playground equipment, with 91.2% reporting a grassy area, 77.7% reporting basketball courts, and 70.6% reporting a blacktop or hard surface area in their outdoor space. Fewer schools not meeting recess requirements reported having a grassy field (*p* =.026) compared to schools meeting recess requirements. Fewer schools with higher Black student enrollment reported having a baseball/softball/soccer field (*p* <.001) or basketball courts (*p* =.024) compared to schools with lower Black student enrollment. The most common equipment provided was playground balls (91.0%), basketballs (88.0%), and soccer balls (80.8%). Fewer schools not meeting reported providing soccer (*p* =.046) and basketballs (*p* =.024). Fewer schools with higher Black enrollment reported providing soccer balls (*p* =.017), footballs (*p* <.001) and allowing students to bring equipment from home (*p* =.002), but more reported providing jump ropes (*p* =.038). The most common space for indoor recess was the classroom (89.4%). A greater percentage of rural schools 17.7%) reported using a multipurpose room for indoor recess compared to urban schools (3.9%, *p* =.003).

**Table 5:** Characteristics of recess from survey

		Total		Meeting recess requirements	Not meeting recess requirements	Urban	Rural	Low Black	High Black	
**Supervision** (***n = 170)***										
*Paras*		106 (62.4%)		89 (61.4%)	16 (66.7%)	62 (60.8%)	44 (64.7%)	80 (65.0%)	26 (55.3%)	
*Classroom teachers*		164 (96.5%)		141 (87.2%)	22 (91.7%)	97 (95.1%)	67 (98.5%)	119 (96.8%)	45 (95.7%)	
*PE Teachers*		31 (18.2%)		24 (16.6%)	6 (25.0%)	16 (15.7%)	15 (22.1%)	25 (20.3%)	6 (12.8%)	
*Administrators*		36 (21.2%)		31 (21.4%)	5 (20.8%)	19 (18.6%)	17 (25.0%)	23 (18.7%)	13 (27.7%)	
*Other*		13 (7.7%)		13 (9.0%)	0 (0%)	9 (8.8%)	4 (5.9%)	10 (8.1%)	3 (6.4%)	
**Reasons for Withholding Recess** (***n = 170)***										
*Student Choice*		29 (17.1%)		27 (18.6%)	2 (8.3%)	15 (14.7%)	14 (20.6%)	21 (17.1%)	8 (17.0%)	
*Sickness*		106 (62.4%)		85 (58.6%)	20 (83.3%)	60 (58.8%)	46 (67.7%)	75 (61.0%)	31 (66.0%)	
*Make up classwork*		45 (26.5%)		35 (24.1%)	10 (41.7%)	22 (21.6%)	23 (33.8%)	**38 (30.9%)**	7 (14.9%)*	
*Punishment*		44 (25.9%)		36 (24.8%)	8 (33.3%)	22 (21.6%)	22 (32.4%)	36 (29.3%)	8 (17.0%)	
*Homework*		10 (5.9%)		8 (5.5%)	2 (8.3%)	6 (5.9%)	4 (5.9%)	**10 (8.1%)**	0 (0%)*	
*Test/Quiz*		18 (10.6%)		13 (9.0%)	5 (20.8%)	9 (8.8%)	9 (13.2%)	**17 (13.8%)**	1 (2.1%)*	
*Student Reward*		19 (11.2%)		14 (9.7%)	5 (20.8%)	9 (8.8%)	10 (14.7%)	16 (13.0%)	3 (6.4%)	
*Other*		55 (32.4%)		52 (35.9%)	3 (12.5%)	37 (36.3%)	18 (26.5%)	38 (30.9%)	17 (36.2%)	
**Outdoor Spaces (** ***n = 170)***										
*Blacktop or hard surface*		120 (70.6%)		106 (73.1%)	13 (54.2%)	74 (72.6%)	46 (67.7%)	88 (71.5%)	32 (68.1%)	
*Grassy field/area*		155 (91.2%)		**135 (93.1%)**	19 (79.2%)*	92 (90.2%)	63 (92.7%)	115 (93.5%)	40 (85.1%)	
*Playground Equipment*		170 (100%)		145 (100%)	24 (100%)	102 (1005)	68 (100%)	123 (100%)	47 (100%)	
*Baseball/softball/soccer field*		65 (38.2%)		55 (37.9%)	9 (37.5%)	44 (43.1%)	21 (30.9%)	**57 (46.3%)**	8 (17.0%)*	
*Basketball court*		132 (77.7%)		115 (79.3%)	16 (66.7%)	77 (75.5%)	55 (80.9%)	**101 (82.1%)**	31 (66.0%)*	
**Equipment (** ***n = 167)***										
*Jump ropes*		71 (42.5%)		63 (44.4%)	8 (33.3%)	40 (40.0%)	31 (46.3%)	**46 (37.7%)**	25 (55.6%)*	
*Playground balls*		152 (91.0%)		130 (91.6%)	22 (91.7%)	93 (93.0%)	59 (88.1%)	113 (92.6%)	39 (86.7%)	
*Soccer balls*		135 (80.8%)		**119 (83.8%)**	16 (66.7%)*	84 (84.0%)	51 (76.1%)	**104 (85.3%)**	31 (68.9%)*	
*Footballs*		111 (66.5%)		97 (68.3%)	14 (58.3%)	66 (66.0%)	45 (67.2%)	**92 (75.4%)**	19 (42.2%)*	
*Basketballs*		147 (88.0%)		**129 (90.9%)**	18 (75.0%)*	91 (91.0%)	56 (83.6%)	111 (91.0%)	36 (80.0%)	
*Hula hoops*		60 (35.9%)		54 (38.0%)	6 (25.05)	33 (33.0%)	27 (40.3%)	40 (32.8%)	20 (44.4%)	
*Can bring equipment from home*		99 (59.3%)		87 (61.3%)	11 (45.8%)	58 (58.0%)	41 (61.2%)	**81 (66.4%)**	18 (40.0%)*	
*Other*		18 (10.8%)		15 (10.6%)	3 (12.5%)	11 (11.0%)	7 (10.5%)	14 (11.5%)	4 (8.9%)	
**Indor Recess Space (o** ***n = 170)***										
*Classroom*		152 (89.4%)		130 (90.0%)	21 (87.5%)	92 (90.2%)	60 (88.2%)	111 (90.2%)	41 (87.2%)	
*Gym*		64 (37.7%)		56 (38.6%)	8 (33.3%)	33 (32.4%)	31 (45.6%)	46 (37.4%)	18 (38.3%)	
*Lunchroom/Stage*		7 (4.1%)		6 (4.1%)	1 (4.2%)	4 (3.9%)	3 (4.4%)	4 (3.3%)	3 (6.4%)	
*Multipurpose Room*		16 (9.4%)		15 (10.3%)	1 (4.2%)	**4 (3.9%)**	12 (17.7%)*	13 (10.6%)	3 (6.4%)	
*Other*		8 (4.7%)		7 (4.8%)	1 (4.2%)	6 (5.9%)	2 (2.9%)	7 (5.7%)	1 (2.1%)	

* *P* <.05 comparing meeting recess requirements to not meeting recess requirements, urban to rural, or low Black to high Black

## Discussion

This study sought to determine school-level implementation of one state’s state-level recess requirement. Compliance with Arkansas’s 40-minute minimum recess requirement was high. When compliance was compared by school characteristics, the only statistically significant difference was schools with higher Black student enrollment were less likely to report complying with the recess requirement. However, three-quarters of schools with higher Black student enrollment were still meeting the 40-minute recess requirement.

In the current study, 99% of schools reported at least 20 min of recess per day, the CDC’s recommended minimum amount of daily recess. These estimates are much higher than previous national recess provision estimates [11], though no national surveillance system currently exists. Based on different secondary data sources, Clevenger et al. estimated that between 65 and 80% of children received 20 or more minutes of daily recess between 2012 and 2016 [11], however these sources of recess compliance data range from administration report at the school and district level to parent report. Additionally, this data was collected prior to the COVID-19 pandemic which likely influenced school policies and practices [34]. The most recent national survey of recess practices was conducted in 2019–2020 and found that 59% of school administrators reported at least 30 min of recess daily, while 88% offered at least 20 min of daily recess [12]. The higher estimates of recess provision in Arkansas are expected, as Arkansas requires 40-minutes of recess, the most recess of any state requirement at the time the current study was conducted. This suggests that higher state recess requirements may lead to higher school recess provision, though the relationship between state policies and school recess implementation may be more complicated in the national setting or depending on the specific policy [22]. Schools meeting recess requirements were more likely to have grassy fields and provide loose equipment, suggesting increased capacity for recess, though additional research is needed to explore the direction of this relationship. The current findings also suggest that state recess policies may create ceiling effects, with only 10% of schools providing more than the required 40-minutes. This may differ with different recess requirements, as 40-minutes is currently twice the 20 min minimum recommended by the CDC [1], so requirements higher than 40 min would be less expected. Anecdotally, some schools have reported reducing recess time when the Arkansas legislation went into effect. Longitudinal and natural experiment studies are needed to understand how recess policies change and influence recess practices at the school level.

Importantly, the current study found that schools with higher Black student enrollment were less likely to meet recess requirements compared to those with lower Black student enrollment, and these disparities remained when adjusting for school economic disadvantage and geography. While we found no statistical difference by region, it is important to note that less than 1% of higher Black enrollment schools are in the Northwest region and 68% of schools with higher Black enrollment schools are in the Southeast region. The previous national study by Tsai et al. [12] found no differences in recess compliance according to school socioeconomic status, racial enrollment, or rurality. Their survey included 21% of schools in rural locations and 9% of schools with a majority of Black students, defined as at least 50% of Black student enrollment, compared to the 25% cut-off used in the current study which may explain the discrepancy in findings. Additionally, data in the current study suggested that the racial disparity may be greater in rural locations, which may indicate why a difference was found in the current study with the larger percentage (40% compared to 21%) of rural schools and overall rural location of Arkansas. Arkansas does not have large urban areas. Future studies in other geographies should explore whether urban and rural disparities in the implementation of recess policy are greater when larger urban areas are included. Additionally, implementation of educational policy implementation among schools primarily serving other health disparate populations such as Hispanic and Indigenous populations should be examined.

There may be several reasons for the disparity in reported recess compliance between schools with higher and lower Black student enrollment. The current study found that schools in compliance had slightly higher academic achievement, which is strongly negatively correlated with economic advantage, but no differences in value-added growth scores which is more independent of poverty status. Due to high correlations among economic status, race and achievement [35], schools with higher Black student enrollment may feel more pressure to improve academics and take as much time as possible for core academic subjects or supporting extracurriculars (e.g., tutoring) and thus provide less recess time. However, schools with higher Black student enrollment were less likely to report withholding recess for academic reasons. Secondly, this current study only examined reported recess policy by principals, other administrators, and school secretaries. Future studies are needed to objectively observe actual recess practices as well as to explore awareness of recess requirements by school personnel and community members in order to advocate for high quality recess policies and practices. Additionally, while we observed a difference in recess compliance between schools by Black student enrollment, this is a cross-sectional study with a risk of Type III error, and other factors may explain the relationship between school racial enrollment and recess compliance. While we did not see a difference in recess compliance between socioeconomic factors as measured by the percent of students receiving FRL, there may be other unmeasured confounders that are driving the differences in compliance. There were additional racial discrepancies that arose in the survey responses indicating potential differences in the quality of recess including differences in recess space and equipment as well as withholding policies. There were reported differences between equipment and spaces by school demographics, and both equipment and playground space has shown to influence physical activity during recess [36]. Longitudinal and mixed-methods studies to discover local factors contributing to recess implementation are needed to better understand the factors influencing these policy decisions.

Increasing awareness of quality recess practices and requirements may be a first step in addressing the gap in recess compliance. The Arkansas recess policy was initiated by community members and parents [37] and recess legislation often has been promoted by parent and community advocates [38]. Thus building capacity through parent teacher organization engagement and community events, may increase recess quality in schools not currently in compliance. However, as the communities most likely experiencing limited recess are often under-resourced, these grassroot initiatives may be less likely to occur and additional top-down support may be needed to ensure all students receive quality recess. Other strategies to reduce the gap include better surveillance of recess policies and providing education about the benefits of recess and being outdoors which have shown to improve—or at minimum not detract from—academics [3]. This might help counter fear of potential unintended consequences of increasing allocation of time to recess. Recess improvements offer both place-based and policy-based educational strategies for improving student outcomes by incorporating local contexts, culture and community experiences into the needs of all students within a community [39], and may be a tangible strategy to engage communities in advocacy for education and health.

This study utilized multiple methods to inclusively capture diverse schools that may not traditionally respond to surveys and was able to obtain recess information from 70% of schools in the state which is higher than previous response rates. In previous studies, Tsai et al. had a 55% response rate on surveys [12] and Clevenger et al. reported 62% participation from districts and 71% participation from 281 participating schools in surveys delivered in-person as part of the School Health Policies and Practices Survey [22]. While there were no differences in school size, race, geography or academic achievement between those included, schools without recess info had higher rates of students receiving FRL potentially suggesting that schools without recess info differ from included schools and may have different recess compliance rates. For schools without information, when contacted by phone, only 13 directly refused to give the information, while the majority either took a message or passed the phone call to the principal to leave a message that was never returned. This could represent a bias from not wanting to discuss recess if they are out of compliance or competing school priorities where recess is not highly valued. Using the multiple methods, we did see some discrepancies in data by source, though minimal. We used a conservative approach when conflicts arose defaulting to meeting guidelines, so actual compliance may be lower than reported. We also observed that schools with online only information were significantly different in being more urban and less likely to meet compliance compared to those surveys or interviews. Future research is needed to better understand the purpose of publicly available bell schedules and whether they are intended to provide complete recess information.

Importantly, the recess time reported in the current study does not reflect regular practice and what dose of recess students receive, nor the quality of recess. It also does not include any waivers received by the schools as defined in the law. However, no participants in the survey or phone conversations mentioned the waivers.

## Conclusions

Schools in Arkansas, a state with a 40-minute daily recess requirement, report high compliance with the state policy. All schools reported offering daily recess, and 90% reported over the CDC’s recommendation for 20 min of daily recess. While longitudinal and natural experiments are needed to see how changes in state recess policies change school recess implementation, this robust study suggests that higher state level recess requirements may increase the amount of recess offered in elementary schools. Implementation was high across schools from urban and rural locations, as well as socioeconomic and demographic school characteristics. However, schools with higher Black student enrollment were less likely to meet the 40-minute recess requirement compared to schools with lower Black student enrollment, and there were differences in reported recess equipment and space. Thus, strategies are needed to ensure all students have access to equal recess opportunities. With the multiple benefits from recess including physical, social and educational development, ensuring equal access to recess through wide-reaching place-based and policy-based strategies may be a step in reducing health and education disparities, especially in regions where disparities are greatest.

## Electronic supplementary material

Below is the link to the electronic supplementary material.

Supplementary Material 1

## Data Availability

The datasets used and analyzed during the current study are available from the corresponding author on reasonable request.

## Abbreviations

CDC: Centers for Disease Control and Prevention
FRL: Students eligible for free-or-reduced-lunch

## References

[1] Centers for Disease C, Prevention. Strategies for supporting recess in elementary schools. Centers for Disease Control and Prevention; 2014.

[2] Bjornsen MA, Perryman KL, Cameron L, Thomas H, Howie EK. The impact of recess on students: a scoping review of developmental outcomes and methodological considerations. J Res Child Educ. 2024;38(4):1–16.

[3] Howie EK, Perryman KL, Moretta J, Cameron L. Educational outcomes of recess in elementary school children: a mixed-methods systematic review. PLoS ONE. 2023;18(11):e0294340. 37992031 10.1371/journal.pone.0294340 PMC10664954

[4] 2018 Physical Activity Guidelines Advisory Committee. 2018 Physical Activity Guidelines Advisory Committee Report,. Washington, DC: U.S. Department of Health and Human Services; 2018.

[5] Clevenger KA, McKee KL, McNarry MA, Mackintosh KA, Berrigan D. Association of recess provision with accelerometer-measured physical activity and sedentary time in a representative sample of 6- to 11-year-old children in the United States. Pediatr Exerc Sci. 2024;36(2):83–90. 37758264 10.1123/pes.2023-0056

[6] Clevenger KA, Belcher BR, Berrigan D. Associations between amount of recess, physical activity, and cardiometabolic traits in U.S. Children. Transl J Am Coll Sports Med. 2022;7(3). 10.1249/tjx.0000000000000202 PMC9531844 36204452

[7] Szecsi T. To have or not to have: recess from an international perspective. Child Educ. 2006;82(4):1.

[8] Aubert S, Barnes JD, Demchenko I, Hawthorne M, Abdeta C, Abi Nader P, et al. Global matrix 4.0 physical activity report card grades for children and adolescents: results and analyses from 57 countries. J Phys Act Health. 2022;19(11):700–28. 36280233 10.1123/jpah.2022-0456

[9] Rodgers CH, Daniels BT, Howie EK. A cross-cultural study of physical activity during recess in Senegal and the United States. Afr J Phys Activity Health Sci (AJPHES). 2024;30(4):632–54.

[10] Van Dyck D, Timmerman C, Hermida A, Pintado R, Cárdenas M, Escandón S, et al. Physical activity during recess in elementary schoolchildren in Belgium and Ecuador: the role of the physical environment at school. J Sports Sci. 2022;40(13):1476–85. 35703158 10.1080/02640414.2022.2086521

[11] Clevenger KA, Dunton GF, Katzmarzyk PT, Pfeiffer KA, Berrigan D. Adherence to recess guidelines in the United States using nationally representative data: implications for future surveillance efforts. J Sch Health. 2023;93(12):1145–55. 37317050 10.1111/josh.13344

[12] Tsai MM, Olarte DA, Hager ER, Cohen JFW, Turner L. Prevalence of recess and supportive practices at a nationwide sample of public elementary schools in the United States. J Sch Health. 2024;94(4):366–73. 37395014 10.1111/josh.13368

[13] Thompson HR, London RA. Not all fun and games: disparities in school recess persist, and must be addressed. Prev Med Rep. 2023;35:102301. 37408995 10.1016/j.pmedr.2023.102301 PMC10319329

[14] Brownson RC, Jones E. Bridging the gap: translating research into policy and practice. Prev Med. 2009;49(4):313–5. 19555708 10.1016/j.ypmed.2009.06.008

[15] Howie EK, Stevick ED. The “ins” and “outs” of physical activity policy implementation: inadequate capacity, inappropriate outcome measures, and insufficient funds. J Sch Health. 2014;84(9):581–5. 25117892 10.1111/josh.12182 PMC4135305

[16] Dills AK, Morgan HN, Rotthoff KW, Recess. Physical education, and elementary school student outcomes. Econ Educ Rev. 2011;30(5):889–900.

[17] National Center for Health Statistics. National Health Interview Survey. 2018. Rockville, MD: Centers for Disease Control and Prevention; 2018.

[18] Skinner ACRS, Skelton JA, Perrin EM, Armstrong SC. Prevalence of obesity and severe obesity in US children, 1999–2016. Pediatrics. 2018;142(3). 10.1542/peds.2017-3459 PMC6109602 29483202

[19] Parrott HM, Cohen LE. Advocating for play: the benefits of unstructured play in public schools. Sch Comm J. 2020;30(2):229–54.

[20] United Nations. Convention on the rights of the child 1989 [Available from: https://www.ohchr.org/en/instruments-mechanisms/instruments/convention-rights-child

[21] Clevenger KA, Perna FM, Moser RP, Berrigan D. Associations between state laws governing recess policy with children’s physical activity and health. J Sch Health. 2022;92(10):976–86. 35266151 10.1111/josh.13157 PMC9458774

[22] Clevenger KA, Lowry M, Perna FM, Berrigan D. Cross-sectional association of state recess laws with district-level policy and school recess provision in the United States. J Sch Health. 2022;92(10):996–1004. 35416309 10.1111/josh.13189

[23] Poulos A, Wilson K, Schulke M, Nam K, Ohri-Vachaspati P, Bai Y, et al. A natural experiment to assess recess frequency on children’s physical activity in Arizona (U.S.) elementary schools. BMC Public Health. 2024;24(1):225. 38238751 10.1186/s12889-023-17605-4 PMC10797942

[24] National Cancer Institute. Classification of laws associated with school students [Available from: https://class.cancer.gov/

[25] Groves RM. Nonresponse rates and nonresponse bias in household surveys. Int J Public Opin Q. 2006;70(5):646–75.

[26] Kern BD, Graber KC, Shen S, Hillman CH, McLoughlin G. Association of school-based physical activity opportunities, socioeconomic status, and third‐grade reading. J School Health. 2018;88(1):34–43. 29224216 10.1111/josh.12581

[27] Arkansas Department of Education. ADE Data Center: Statewide Information System Reports 2024 [Available from: https://adedata.arkansas.gov/statewide/Default.aspx

[28] Enrollment by Grade by School. ADE Data Center. 2024. Available from: https://adedata.arkansas.gov/statewide/ReportList/Schools/EnrollmentByGrade.aspx

[29] Goldsmith PA. Schools’ racial mix, students’ optimism, and the Black-White and Latino-White achievement gaps. Sociol Educ. 2004;77(2):121–47.

[30] National Center for Education Statistics. Racial/Ethnic enrollment in public schools. U.S. Department of Education, Institute of Education Sciences; 2024.

[31] Martin MA, Thomas T, Adler GJ Jr., Kreager DA. Are feminine body weight norms different for black students or in black schools? Girls’ weight-related peer acceptance across racialized school contexts. J Health Soc Behav. 2020;61(2):239–58. 32506964 10.1177/0022146520920599 PMC8059344

[32] Federal Office of Rural Health Policy. List of Rural Counties and Designated Eligible Census Tracts in Metropolitan Counties: Updated Census 2010/0 MB Bulletin No. 20–01 2021.

[33] Office for Education Policy. University of Arkansas,; [Available from: https://oep.uark.edu/data/school-recognition-and-letter-grades/

[34] World Bank. The COVID-19 pandemic: shocks to education and policy responses. World Bank; 2020.

[35] Battle J, Lewis M. The increasing significance of class: the relative effects of race and socioeconomic status on academic achievement. J Poverty. 2002;6(2):21–35.

[36] Delidou E, Matsouka O, Nikolaidis C. Influence of school playground size and equipment on the physical activity of students during recess. Eur Phy Educ Rev. 2016;22(2):215–24.

[37] Howell C. More-recess-time law playing out at Arkansas schools; North little rock district opts to lengthen class day. Ark Democrat Gaz. 2019.

[38] Tandon PS, Westerlind L, McCleery J. Advocacy for equitable recess in Washington state. Pediatrics. 2024;153(5). 10.1542/peds.2023-064226 38572558

[39] Yemini M, Engel L, Ben Simon A. Place-based education– a systematic review of literature. Educ Rev. 2025;77(2):640–60.

