# Supplementary material for: Elementary school compliance with a state recess minimum requirement by racial and geographic factors: a cross-sectional study
Source: Int J Behav Nutr Phys Act. 2025 Mar 28;22:37. doi: 10.1186/s12966-025-01730-x (PMC11954336; doi:10.1186/s12966-025-01730-x)
Supplement: Supplementary file 1 — Supplementary Material 1 [file 12966_2025_1730_MOESM1_ESM.docx]

**Supplemental Content**

**Supplemental Table 1:** School characteristics compared by meeting recess requirements for all schools including those with online only recess information, mean(SD) or n(%)

|  | Meeting recess recs (n=316) | Not meeting recess recs (n=68) | p-value |
| --- | --- | --- | --- |
| School Enrollment | 399.2 (156.6) | 412.5 (159.9) | .527 |
| Grades in School |  |  | .146 |
| *Lower* | 25 (7.9%) | 3 (4.4%) |  |
| *Upper* | 25 (7.9%) | 10 (14.7%) |  |
| *Mixed* | 266 (84.2%) | 55 (80.1%) |  |
| Enrollment by Race |  |  |  |
| *% Black* | 17.1 (24.9) | 30.0 (32.9) | **<.001** |
| *% Hispanic* | 13.1 (14.5) | 11.5 (16.3) | .438 |
| *% White* | 60.9 (27.9) | 51.3 (32.5) | **.013** |
| *% Minority* ^a^ | 38.0 (27.4) | 47.6 (32.9) | **.012** |
| Schools with high Black enrollment (%) | 78 (24.7%) | 27 (39.7%) | **.012** |
| Students receiving FRL (%) ^a^ | 63.1 (20.0) | 67.1 (2.4) | .133 |
| Schools in Rural Counties (%) | 124 (39.2%) | 23 (33.8%) | .404 |
| Region |  |  | .090 |
| *1 -Northwest* | 123 (38.9%) | 18 (26.5%) |  |
| *2 - Northeast* | 56 (17.7%) | 17 (25.0%) |  |
| *3 - Central* | 94 (29.8%) | 20 (29.4%) |  |
| *4 – Southwest* | 30 (9.5%) | 6 (8.8%) |  |
| *5 - Southeast* | 13 (4.1%) | 7 (10.3%) |  |
| Charter School ^a^ | 10 (3.2%) | 4 (5.9%) | .286 |
| ESSA Index ^a^ | 68.4 (7.6) | 66.5 (7.4) | .056 |
| Weighted Achievement ^a^ | 58.0 (14.9) | 53.9 (15.2) | **.043** |
| Growth Score ^a^ | 80.8 (3.3) | 80.6 (2.8) | .622 |
| School Quality ^a^ | 51.5 (10.3) | 48.8 (10.8) | **.049** |

^a^ 8 schools did not have school achievement (including %FRL, and %minority) from 2022-2023 OEP (n=3 with recess info, 5 without recess info).

**Supplemental Table 2**: Logistic regression results of odds of meeting recess requirements by geographic and racial school characteristics with **Black student enrollment as a continuous variable**

|  | **OR** | **95%CI** | **p** |
| --- | --- | --- | --- |
| All available info (n=384) | | | |
| Race (% Black) | 0.30 | 0.10, 0.87 | .027 |
| Rural | **2.01** | **0.95, 4.24** | **.067** |
| Region |  |  | .188 |
| *2 - Northeast* | **0.48** | **0.20, 1.12** | **.088** |
| *3 - Central* | 1.19 | 0.54, 2.62 | .663 |
| *4 – Southwest* | 0.72 | 0.23, 2.21 | .565 |
| *5 - Southeast* | 0.32 | 0.08, 1.26 | .103 |
| Removing online only (n=358) | | | |
| Race (% Black) | **0.13** | **0.04, 0.44** | **.001** |
| Rural | 1.60 | 0.71, 3.56 | .254 |
| Region |  |  | .101 |
| *2 - Northeast* | 0.58 | 0.22, 1.48 | .252 |
| *3 - Central* | 2.30 | 0.84, 6.24 | .103 |
| *4 – Southwest* | 0.81 | 0.25, 2.66 | .726 |
| *5 - Southeast* | 0.59 | 0.13, 2.72 | .499 |

**Supplemental Table 3**: Logistic regression results of odds of meeting recess requirements by geographic and racial school characteristics All available info (n=381)

|  | **OR** | **95%CI** | **p** |
| --- | --- | --- | --- |
| Model 1 | | | |
| Race (High Black) | 0.67 | 0.35, 1.29 | .234 |
| Rural | **2.19** | **1.04, 4.60** | **.038** |
| Region |  |  | .087 |
| *2 - Northeast* | **0.42** | **0.18, 0.98** | **.046** |
| *3 - Central* | 1.02 | 0.47, 2.22 | .968 |
| *4 – Southwest* | 0.61 | 0.20, 1.87 | .389 |
| *5 - Southeast* | 0.21 | 0.06, 0.78 | .020 |
| Model 2 | | | |
| School enrollment | 1.00 | 0.997, 1.001 | .506 |
| %FRL | 1.00 | .98, 1.01 | .374 |
| Race (High Black) | 0.75 | 0.36, 1.54 | .432 |
| Rural | **2.39** | **1.11, 5.14** | **.026** |
| Region |  |  | .087 |
| *2 - Northeast* | **0.40** | **0.17, 0.98** | **.044** |
| *3 - Central* | 0.98 | 0.45, 2.15 | .958 |
| *4 – Southwest* | 0.59 | 0.19 ,1.81 | .354 |
| *5 - Southeast* | **0.20** | **0.05, 0.75** | **.017** |
